# Construction and Bioinformatics Analysis of ceRNA Regulatory Networks in Idiopathic Pulmonary Fibrosis

**DOI:** 10.1007/s10528-024-10853-y

**Published:** 2024-06-13

**Authors:** Menglin Zhang, Xiao Wu, Honglan Zhu, Chenkun Fu, Wenting Yang, Xiaoting Jing, Wenqu Liu, Yiju Cheng

**Affiliations:** 1 Department of Respiratory and Critical Care Medicine, The Affiliated Hospital of Guizhou Medical University, Guiyang, 550004 China; 2 Department of Respiratory and Critical Care Medicine, The Fourth People’s Hospital of Guiyang, Guiyang, 550002 China; 3 Department of Clinical Medicine, Guizhou Medical University, Guiyang, 550004 China; 4 Department of Critical Care Medicine, The Second People’s Hospital of Guiyang, Guiyang, 550004 China; 5 Department of Respiratory and Critical Care Medicine, People’s Hospital of Anshun City Guizhou Province, Anshun, 561000 China; 6 Department of Respiratory and Critical Care Medicine, Guiyang Public Health Clinical Center, Guiyang, 550002 China

**Keywords:** Pulmonary fibrosis, ceRNA, lncRNA, miRNA, Differential expression analysis

## Abstract

Idiopathic pulmonary fibrosis (IPF) is a chronic, progressive form of pulmonary fibrosis of unknown etiology. Despite ongoing research, there is currently no cure for this disease. Recent studies have highlighted the significance of competitive endogenous RNA (ceRNA) regulatory networks in IPF development. Therefore, this study investigated the ceRNA network associated with IPF pathogenesis. We obtained gene expression datasets (GSE32538, GSE32537, GSE47460, and GSE24206) from the Gene Expression Omnibus (GEO) database and analyzed them using bioinformatics tools to identify differentially expressed messenger RNAs (DEmRNAs), microRNAs (DEmiRNAs), and long non-coding RNAs (DElncRNA). For DEmRNAs, we conducted an enrichment analysis, constructed protein–protein interaction networks, and identified hub genes. Additionally, we predicted the target genes of differentially expressed mRNAs and their interacting long non-coding RNAs using various databases. Subsequently, we screened RNA molecules with ceRNA regulatory relations in the lncACTdb database based on the screening results. Furthermore, we performed disease and functional enrichment analyses and pathway prediction for miRNAs in the ceRNA network. We also validated the expression levels of candidate DEmRNAs through quantitative real-time reverse transcriptase polymerase chain reaction and analyzed the correlation between the expression of these candidate DEmRNAs and the percent predicted pre-bronchodilator forced vital capacity [%predicted FVC (pre-bd)]. We found that three ceRNA regulatory axes, specifically KCNQ1OT1/XIST/NEAT1-miR-20a-5p-ITGB8, XIST-miR-146b-5p/miR-31-5p- MMP16, and NEAT1-miR-31-5p-MMP16, have the potential to significantly affect IPF progression. Further examination of the underlying regulatory mechanisms within this network enhances our understanding of IPF pathogenesis and may aid in the identification of diagnostic biomarkers and therapeutic targets.

## Introduction

Idiopathic pulmonary fibrosis (IPF) is a diffuse lung disease characterized by progressive lung injury and fibrosis, significantly impairing lung structure and function (King et al. 2011). The exact cause of IPF remains unknown, and its prognosis is generally poor, with a median survival rate of only 2–3 years (Kreuter et al. 2017). The diagnosis of IPF relies on imaging techniques and histopathology, specifically identifying usual interstitial pneumonia manifestations (American Thoracic Society 2000). Currently, the available therapeutic options for IPF are limited, and nintedanib and pirfenidone are the only approved treatments. However, these drugs can only delay disease progression and provide limited lung function improvement without curing IPF (Dempsey et al. 2021). Challenges in diagnosing and treating IPF stem from our incomplete understanding of the disease and the absence of precise biomarkers and therapeutic targets. Therefore, there is an urgent need to develop novel diagnostic and therapeutic approaches. Recent advances in high-throughput sequencing technology have shed light on the significant role of non-coding RNAs (ncRNAs) in the pathogenesis of IPF, offering new insights into the disease mechanisms and potential targets for therapeutic intervention.

**Fig. 1:**
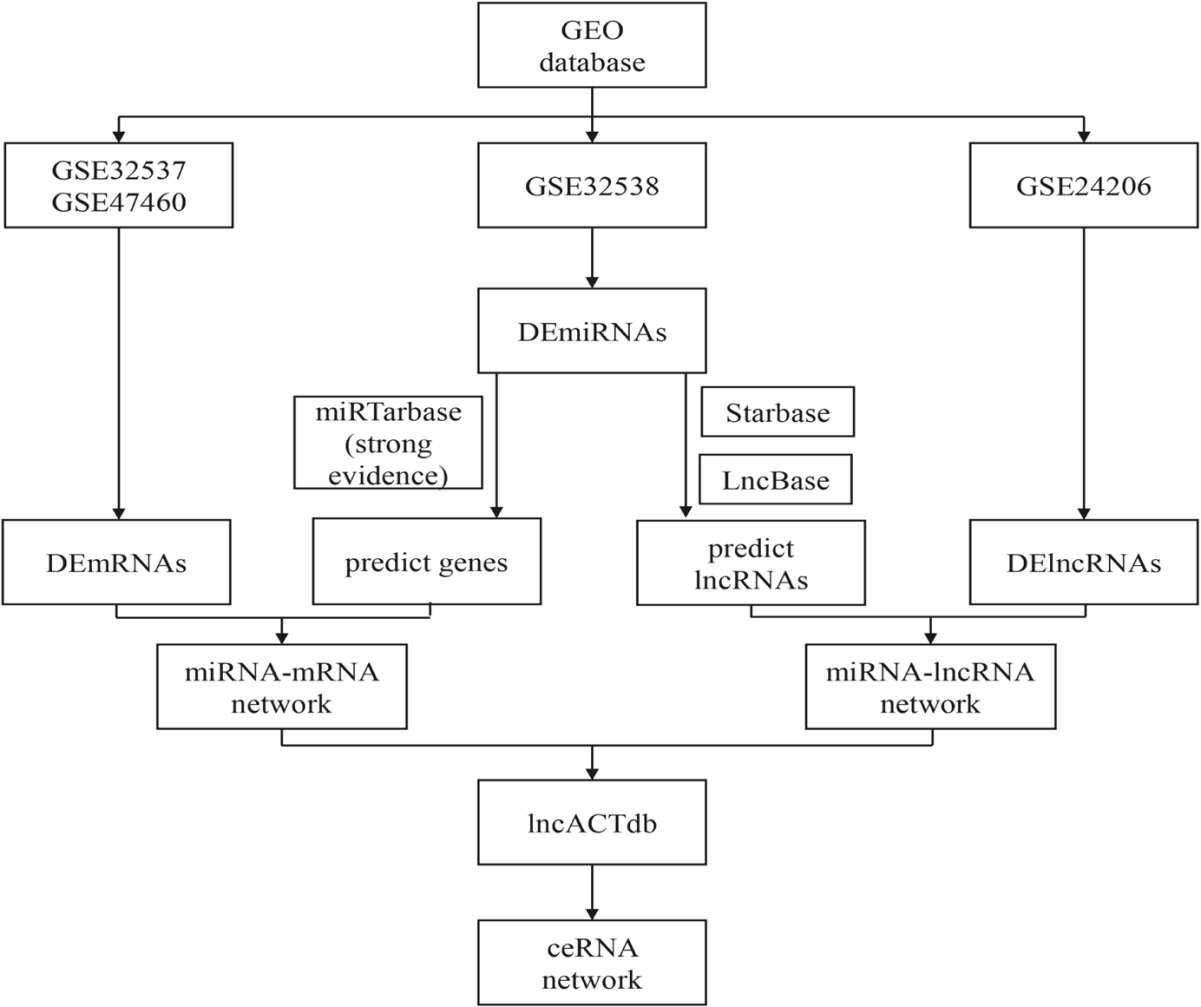
The workflow chart of our study

ncRNAs such as microRNAs (miRNAs) and long non-coding RNAs (lncRNAs) are transcribed from the human genome but do not produce proteins (Kaikkonen et al. 2011). However, lncRNAs have been identified as significant disease regulators, affecting the development and treatment of various diseases by controlling gene expression and cellular processes (Morris and Mattick 2014). Specifically, lncRNAs regulate gene expression at the transcriptional and translational levels through different mechanisms, whereas miRNAs primarily regulate the expression of protein-coding genes by inhibiting messenger RNAs (mRNAs) (Bartel 2018). A growing body of research has shown that abnormally expressed ncRNAs play crucial roles in the pathogenesis of IPF (Hadjicharalambous and Lindsay 2020; Zhang et al. 2021a). For instance, miRNAs, such as miR-21 and miR-33, are abnormally expressed in the lung tissues of patients with IPF and influence disease progression by regulating fibroblast activation and inflammation (Ahangari et al. 2023; Liu et al. 2010). Another study has confirmed that lncRNA MALAT1 was highly expressed in patients with IPF and affected the development of IPF by regulating gene expression, inflammation, and other pathways (Lai et al. 2022). Additionally, studies have identified complex regulatory interactions among ncRNA molecules. lncRNAs can interact with miRNAs as competing endogenous RNAs (ceRNAs) or function as reservoirs, thereby affecting gene expression (Zhang et al. 2021c). The ceRNA mechanism is particularly important in the pathogenesis of IPF, in which RNA molecules, including mRNAs, miRNAs, and lncRNAs, compete for the same miRNA-binding sites, thereby influencing their expression levels (Song et al. 2014; Hadjicharalambous et al. 2018). Experiments have demonstrated that certain lncRNAs can act as ceRNAs, sequester miRNAs, and affect the expression of target genes, ultimately leading to IPF. Song et al. showed that lncRNA H19 binds to miR-196a through a ceRNA mechanism and increases the expression of COL1A1, resulting in pulmonary fibrosis (Lu et al. 2018). However, the specific regulatory mechanisms of lncRNAs, miRNAs, and mRNAs in the development of IPF through the ceRNA mechanism are still unclear. Further studies are needed to uncover how the molecules in these networks affect cellular functions and signaling pathways. In addition, current studies on IPF often rely on cell lines or animal models that may not fully replicate the complex pathology of IPF in humans. Therefore, additional human lung tissue specimens must be collected to develop more accurate models. Furthermore, most studies lack clinical value assessment and validation of abnormally expressed ncRNAs, which may overlook important insights into the pathogenesis of IPF and limit the development of more effective therapeutic approaches. Hence, in this study, we used high-throughput sequencing technology and bioinformatic analysis to identify abnormally expressed lncRNAs, miRNAs, mRNAs, and their interacting ceRNA networks in IPF. Disease, functional, and pathway enrichment analyses were conducted to validate the gene expression of ncRNA molecules in the networks. We analyzed the correlation between these networks and human clinical features to identify ceRNA networks with significant regulatory roles in IPF. In conclusion, we examined three ceRNA regulatory axes. The workflow employed in our study is depicted in Fig. 1. This study establishes a solid theoretical foundation for future functional biology experiments. Additionally, the identification of aberrant ncRNA molecules and their interaction networks in IPF provides valuable insights into disease pathogenesis and facilitates the development of novel diagnostic and therapeutic strategies.

## Materials and Methods

### Data Sources

We searched the Gene Expression Omnibus (GEO) genomics data repository (http://www.ncbi.nlm.nih.gov/geo) to identify gene expression datasets relevant to IPF. The search was performed using the keywords “idiopathic pulmonary fibrosis,” “Homo sapiens,” and “Expression profiling by array” or “Non-coding RNA profiling by array.” Following a systematic review, two mRNA expression datasets (GSE32537 and GSE47460) were selected and downloaded. GSE32537 and GSE47460 were based on the GPL6244 and GPL6480 datasets, respectively. Additionally, one lncRNA expression dataset (GSE24206) was downloaded based on GPL570. One miRNA expression dataset (GSE32538) was selected and downloaded based on GPL8786. The specific dataset information is presented in Table 1.

**Table 1:** Details of miRNA, mRNA and lncRNA datasets of patients with IPF

Type	GEO accession	Platform	Sample organism	Experiment type	Samples (lung tissues), n	
					IPF patients Controls	
mRNA mRNA	GSE32537	GPL6244	Homo sapiens	Expression profiling by array	119	50
	GSE47460	GPL6480	Homo sapiens	Expression profiling by array	38	17
miRNA	GSE32538	GPL8786	Homo sapiens	Non-coding RNA profiling by array	106	50
lncRNA	GSE24206	GPL570	Homo sapiens	Expression profiling by array	17	6

### Differentially Expressed Genes (DEG) Identification

The GEO2R tool, available at http://www.ncbi.nlm.nih.gov/geo/geo2r, was used to identify DEGs between patients with IPF and healthy control tissues. |log fold change|> 1 and adjusted P-value < 0.05 were considered statistically significant.

### Enrichment analysis of the Kyoto Encyclopedia of Genes and Genomes (KEGG) and Gene Ontology (GO) for DEGs

The KEGG database is a valuable resource for elucidating the overarching characteristics and effects of biological systems (Kanehisa et al. 2016). GO is a prominent bioinformatics tool that facilitates gene annotation based on its functional roles in biological processes (BP), molecular functions (MF), and cellular components (CC) (Gene Ontology Consortium 2015). To identify the distinctive attributes of DEGs, the Metascape platform was employed (Zhou et al. 2019), with specific screening criteria of a minimum overlap equal to 3 and minimum richness equal to 1.5. Statistical significance was set at 0.01.

### Construction of Protein–Protein Interaction (PPI) network and screening of hub genes

The filtered DEGs were submitted to the STRING database (http://www.bork.embl-heidelberg.de/STRING/) for further analyses (von Mering et al. 2003). PPI pairs with a combined score greater than 0.4 were extracted. The degrees of all nodes were calculated using the cytoHubba plugin in Cytoscape version 3.7.2 (http://www.cytoscape.org/). In this experiment, the genes with the 10 highest degree values were identified as hub genes.

### Establishment of the ceRNA Network

The construction of this network involved the following three steps. First, an mRNA-miRNA network was constructed by identifying DEmiRNAs from the GSE32538 dataset and predicting their downstream target genes using the miRTarBase database (Huang et al. 2020)**.** The predicted genes were then compared with DEmRNAs from the GSE32537 and GSE47460 datasets to identify candidate target genes. A miRNA-mRNA regulatory network was established by considering the regulatory relationships between miRNAs and mRNAs. Second, a miRNA-lncRNA network was constructed using the Starbase (Li et al. 2014) and Lncbase databases (Karagkouni et al. 2020) to predict lncRNAs that may interact with the DEmiRNAs. These predicted lncRNAs were then compared with DElncRNAs from the GSE24206 dataset to obtain a list of candidate target lncRNAs through intersection analysis. Finally, candidate miRNA-mRNA and miRNA-lncRNA networks were screened for ceRNA networks using the lncACTdb database (Wang et al. 2015, 2019, 2022). The intersection of DEmRNAs, mRNAs, and predicted lncRNAs was integrated and processed using the Cytoscape software.

### Animal Model

Male C57BL/6J mice were procured from SiPeiFu Biotechnology (http://www.spfbiotech.com). Following a one-week acclimatization period, 12 mice were randomly assigned to two groups using a random number table, with six mice in each group. The groups were designated as control [phosphate-buffered saline (PBS)] and bleomycin (BLM) groups. On the day of modeling, all mice were intraperitoneally injected with pentobarbital sodium (50mg/kg) to induce anesthesia. Upon achieving anesthesia, mice in the BLM group were administered an intratracheal injection of 1.5 U/kg of BLM dissolved in 50 μL of PBS, while the PBS group received an equivalent volume of PBS solution. After 28 days, humane euthanasia was performed on all mice by intraperitoneal injection of pentobarbital sodium (150mg/kg), and lung tissues were collected for further experimentation. The study was conducted in accordance with the Declaration of Helsinki and approved by the Animal Experimental Inspection Form of Guizhou Medical University (number:12304822).

### Cell Culture

The human type II alveolar epithelial (A549) and mouse embryonic fibroblast (3T3) cell lines were obtained from Procell (China) and cultured according to the manufacturer’s instructions. A549 cells were cultured in Ham’s F-12 K medium (Gibco, Thermo Fisher Scientific, USA), whereas 3T3 cells were cultured in Dulbecco’s Modified Eagle’s medium (Gibco, Thermo Fisher Scientific, USA). Both media were supplemented with 10% fetal bovine serum (Gibco, Thermo Fisher Scientific, USA) and 1% penicillin–streptomycin. The cells were maintained at 37°C and 5% CO_2_. The transforming growth factor beta 1 (TGF-β1) treatment group was exposed to TGF-β1 (10 ng/mL; MedChemExpress, USA) to induce lung fibroblast activation, while the control group received an equivalent volume of solvent. After 72 h of culture, the cells were harvested.

### Quantitative Real-Time Reverse Transcriptase Polymerase Chain Reaction (qRT-PCR)

Total RNA was extracted from 3T3 cells, A549 cells, and mouse lung tissue using the TRIzol reagent (Takara, Japan) according to the manufacturer’s instructions. Subsequently, complementary DNA was synthesized using a Primer Script RT kit (Takara, Japan). Real-time PCR was performed using the Quant Studio 1 real-time PCR system (Thermo Fisher Scientific, USA) and TB Green Premix Ex TaqTM kit (Takara, Japan). Glyceraldehyde-3-phosphate dehydrogenase was used as a stable internal control for gene expression analysis in lung tissues during pulmonary fibrosis. Primer sequences used in the experiments are listed in Tables 2 and 3. Primer sequences for the mRNAs were obtained from Sangon Biotech (Shanghai, China).

**Table 2:** Human PCR primers

Gene name	Forward primer (5ʹ–3ʹ)	Reverse primer (5ʹ–3ʹ)
PDGRRA	ACGGTCTTGGAAGTGAGCAGTG	GCTACATCTGGGTCTGGCACATAG
TRIM29	GCCGCTTCACCAAGGAGACC	AGAGTTCTGAATGCTGGAGGAGTAC
SFRP2	CCTGGAGACCAAGAGCAAGACC	CTTTGAGCCACAGCACCGATTTC
PEBP4	TTGGACAATGAGGCTGGTCACAG	GGCTGTTCTCATCCTCGTCTCC
MMP16	GAAGGACACAGCCCACCACCAGATG	CCAAGATGCAGGGAATGACAATAGC
FGG	AGAGTGGAACTGGAAGACTGGAATG	TTAGGCGGTACTTGTCAGCTTCAG
CLDN1	AGGTACGAATTTGGTCAGGCTCTC	GGGACAGGAACAGCAAAGTAGGG
ACLS1	CATGCGAAGTGAGCCTGTTGC	GTTCCTCAAACGACCCTTCAAATCC
ITGB8	TGTCTGTGAAAAGTCATATCGGATGG	CACTATGCCTGCCAATTTGCTATC
COL3A1	CTTCTCTCCAGCCGAGCTTC	TGTGTTTCGTGCAACCATCC
GAPDH	CTGACTTCAACAGCGACACC	CC CTGTTGCTGTAGCCAAAT

*PCR* polymerase chain reaction

**Table 3:** Mouse PCR primers

Gene name	Forward primer (5'-3')	Reverse primer (5'-3')
PDGRRA	GACGGATGAGAGTGAGATCGAAGG	TGGTGCGGCAAGGTATGATGG
TRIM29	GGAGAAGCAGAAGGAGGAAGTACG	TCATCCAGAGCATCCACGATCAC
SPRY4	GCAGCAGTAGCAGCACTTCC	TGAGGTCCAGCGGCTTACAG
SFRP2	TGCTGGAGTGCGACCGTTTC	GATGTCGTTGTCGTCCTCATTCTTG
PEBP4	CACTTTGCTTGAGCCTCCTTGC	GGTTTCTCTCCTCCAGGTTTCCC
MMP16	TCGCATTCAGCTCTGGAAGAAGG	GCTCCGTTCCGCAGACTGTAG
FGG	TGGTTTCGACGACGGCATTATTTG	ATGTGATGCTGCTGTCCTTCTCC
CLDN1	GTGTCCTACTTTCCTGCTCCTGTC	AGAAGGTGTTGGCTTGGGATAAGG
ACLS1	CCTGGACGACTTGTTGAAACTTGG	TCTTCGCCTTCAGTGTTGGAGTC
ITGB8	CCAGTGCTTTAGTGGCTGGG	GGCCCTTGGAGTTGACACAG
COL3A1	CATGCATAAATGCCAGCCCC	AGGAGGGCCATAGCTGAACT
GAPDH	GGAGAGTGTTTCCTCGTCCC	ATG AAG GGGTCGTTGATGGC

### Statistical Analysis

The 2^−ΔΔCt^ method was employed to determine the relative expression levels of the relevant genes. The obtained data are presented as mean ± standard error of the mean and analyzed using GraphPad PRISM 9.5.0 software (USA). Differences between the two groups were determined using a t-test. p < 0.05 was regarded as statistically significant. Correlation among the candidate DEmRNAs was evaluated using Pearson's correlation analysis.

## Results

### Identification of DEmRNAs

**Fig. 2:**
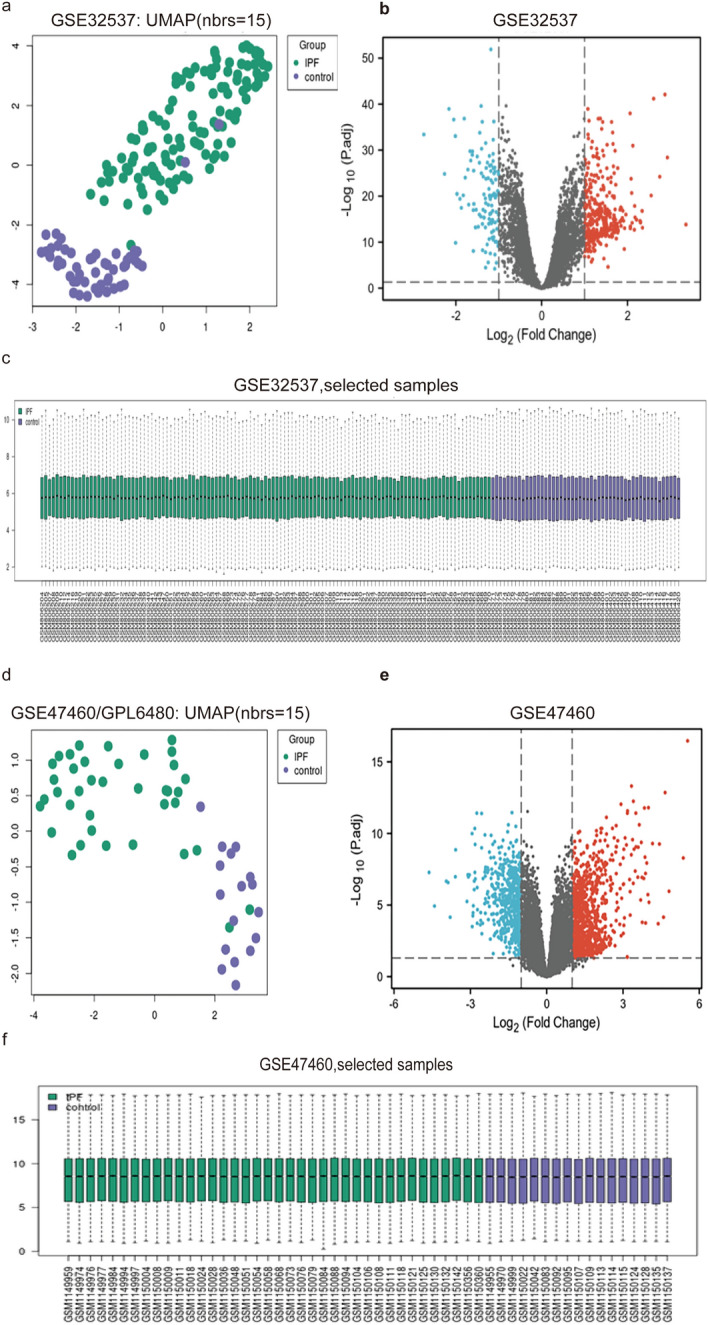
Visual presentation of differential gene screening results in the GSE32537 and GSE47460 dataset. **a** and **d** UMAP plot. **b** and **e** Volcano plots. **c** and **f** processed data boxplots. *UMAP* uniform manifold approximation and projection)

After conducting a reanalysis of the two gene expression profiles, it was determined that GSE32537 exhibited 313 upregulated and 118 downregulated genes with significant differences in their expression. Volcano plots, uniform manifold approximations and projections (UMAPs), and data boxplots were used to visually represent these findings (Fig. 2a–c). Similarly, GSE47460 displayed 826 upregulated and 544 downregulated genes (Fig. 2d–f). Subsequently, bioinformatic tools were used to identify common DEmRNAs between the GSE32537 and GSE47460 datasets. The analysis revealed 176 upregulated and 60 downregulated genes, as shown in Fig. 4 (a, b, c). The demographic characteristics of the GSE47460 dataset are presented in Table 4.

**Table 4:** Demographic data for subjects of GSE47460

Characteristic	Levels	IPF	Normal	p
n		38	17	
Sex, n (%)	Female	9 (16.4%)	8 (14.5%)	0.156
	Male	29 (52.7%)	9 (16.4%)	
Smoker, n (%)	Ever	23 (41.8%)	9 (16.4%)	0.87
	Never	15 (27.3%)	8 (14.5%)	
age, mean ± SD		62.76 ± 8.72	62.7 ± 10.84	0.983
%predicted DLCO, median (IQR)		62.76 ± 8.72	88 (84,103)	< 0.001
%predicted FEV1 (pre-bd), median (IQR)		77.5 (51.75, 82.75)	99 (91,104)	< 0.001
%predicted FVC (pre-bd), mean ± SD		64.34 ± 17.85	93.82 ± 13.63	< 0.001

*IPF* idiopathic pulmonary fibrosis, *FVC* forced vital capacity, *FEV1* forced expiratory volume in the first second, *DLCO* diffusing capacity of the lungs for carbon monoxide, *pre-bd* pre-bronchodilator

### Identification of DEmiRNAs

**Fig. 3:**
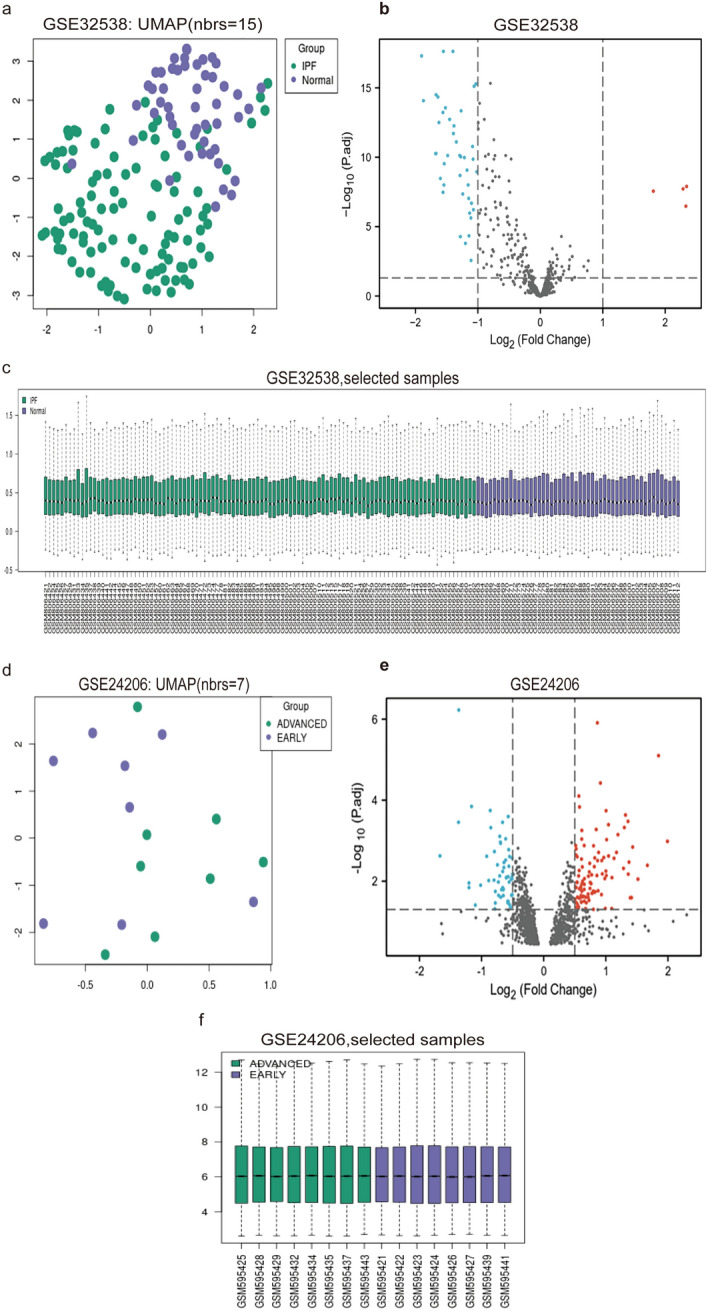
Visual presentation of differential gene screening results in the GSE32538 and GSE24206 dataset. **a** and **d** UMAP plot. **b** and **e** Volcano plots. **c** and **f** processed data boxplots. *UMAP* uniform manifold approximation and projection)

Four genes were identified as being upregulated, whereas 42 miRNAs were found to be downregulated in the GSE32538 dataset. Volcano plots, UMAPs, and data boxplots were employed (Fig. 3a–c).

### Identification of DElncRNAs

We identified 157 upregulated and 79 downregulated lncRNAs in the GSE24206 dataset. To visually represent our findings, we utilized Volcano plots, UMAPs, and data boxplots, as shown in Fig. 3d–f.

### Identification of Hub Genes and Construction of PPI Network of DEmRNAs

**Fig. 4:**
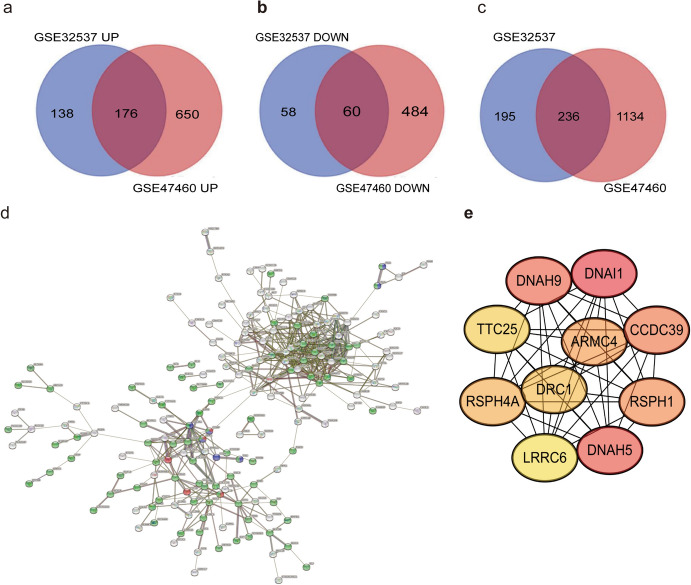
Venn diagrams of the DEmRNAs, **a** cross areas indicate the upregulated DEmRNAs, **b** cross areas indicate the downregulated DEmRNAs, and **c** cross areas indicate the common DEmRNAs, Enrichment analysis, PPI networks, and hub genes about DEmRNAS. **d** Construction of PPI network of DEmRNAs. **e** Identified hub genes. (*DEmRNA* differentially expressed mRNAs, *PPI* protein–protein interaction, *KEGG* Kyoto Encyclopedia of Genes and Genomes)

The MCODE plugin for Cytoscape was used to identify densely connected regions within the PPI network. The resulting networks are shown in Fig. 4d. The top 10 genes that exhibited the highest number of interactions, referred to as hub genes, were identified from these networks. These hub genes included DNAH9, DNAI1, TTC25, ARMC4, CCDC39, RSPH4A, DRC1, RSPH1, LRRC6, and DNAH5, as shown in Fig. 4e.

### Construction of ceRNA Network

**Fig. 5:**
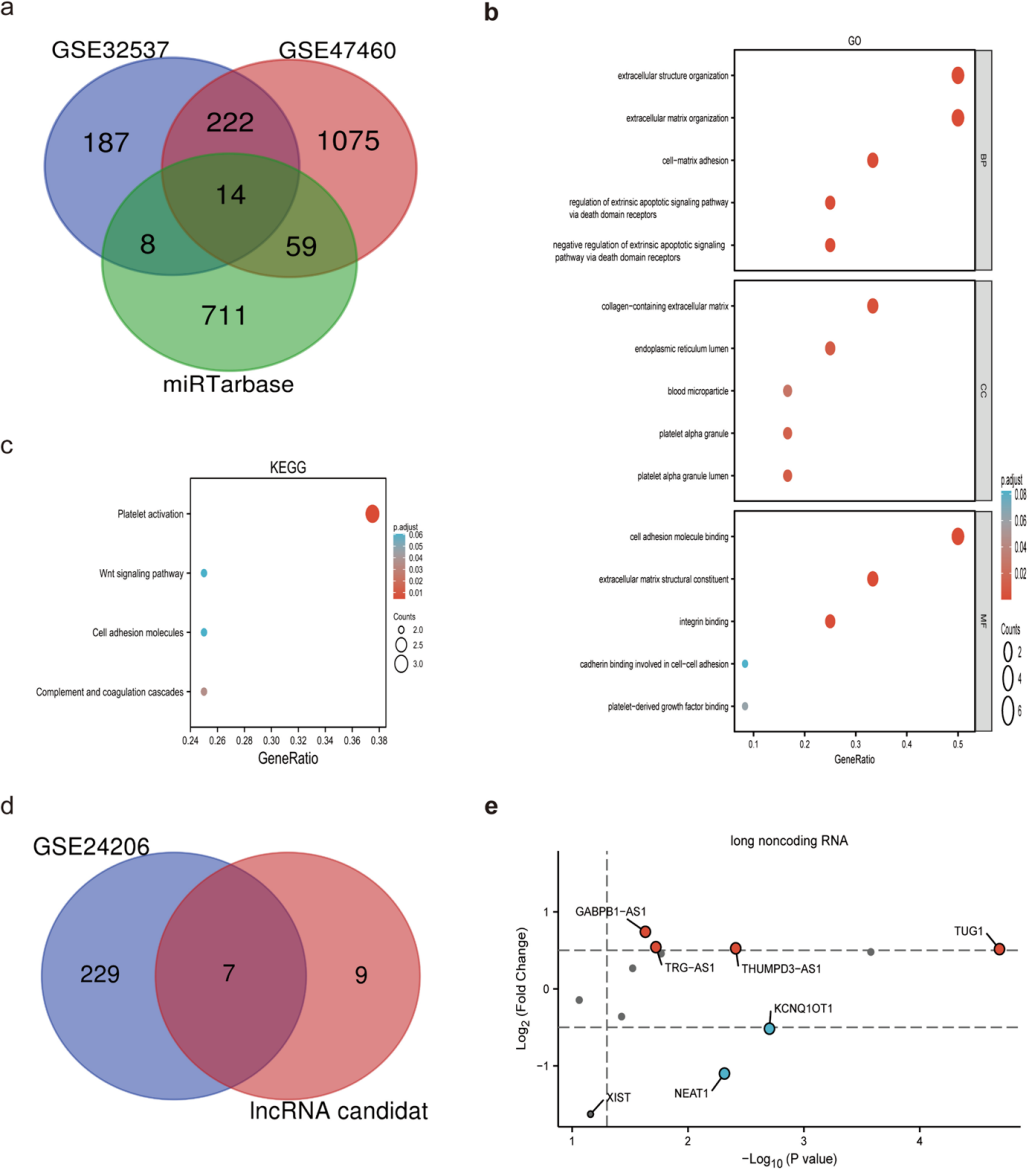
miRNA-mRNA network. **a** Cross areas indicate the candidate DEmRNAs in the miRNA-mRNA network. **b** and **c** Bubble plot of BP, CC, MF and KEGG pathway analysis for candidate DEmRNAs in the miRNA-mRNA network. **d** Cross areas indicate the candidate DELncRNAs in the miRNA-lncRNA network. **e** The Volcano plots of the candidate DElncRNAs. (*miRNA* microRNA, *mRNA* messenger, *BP* biological processes, *CC* cellular components, *MF* molecular functions, *KEGG* Kyoto Encyclopedia of Genes and Genomes, *lncRNA* long non-coding RNA, *DELncRNA* differentially expressed lncRNA)

**Fig. 6:**
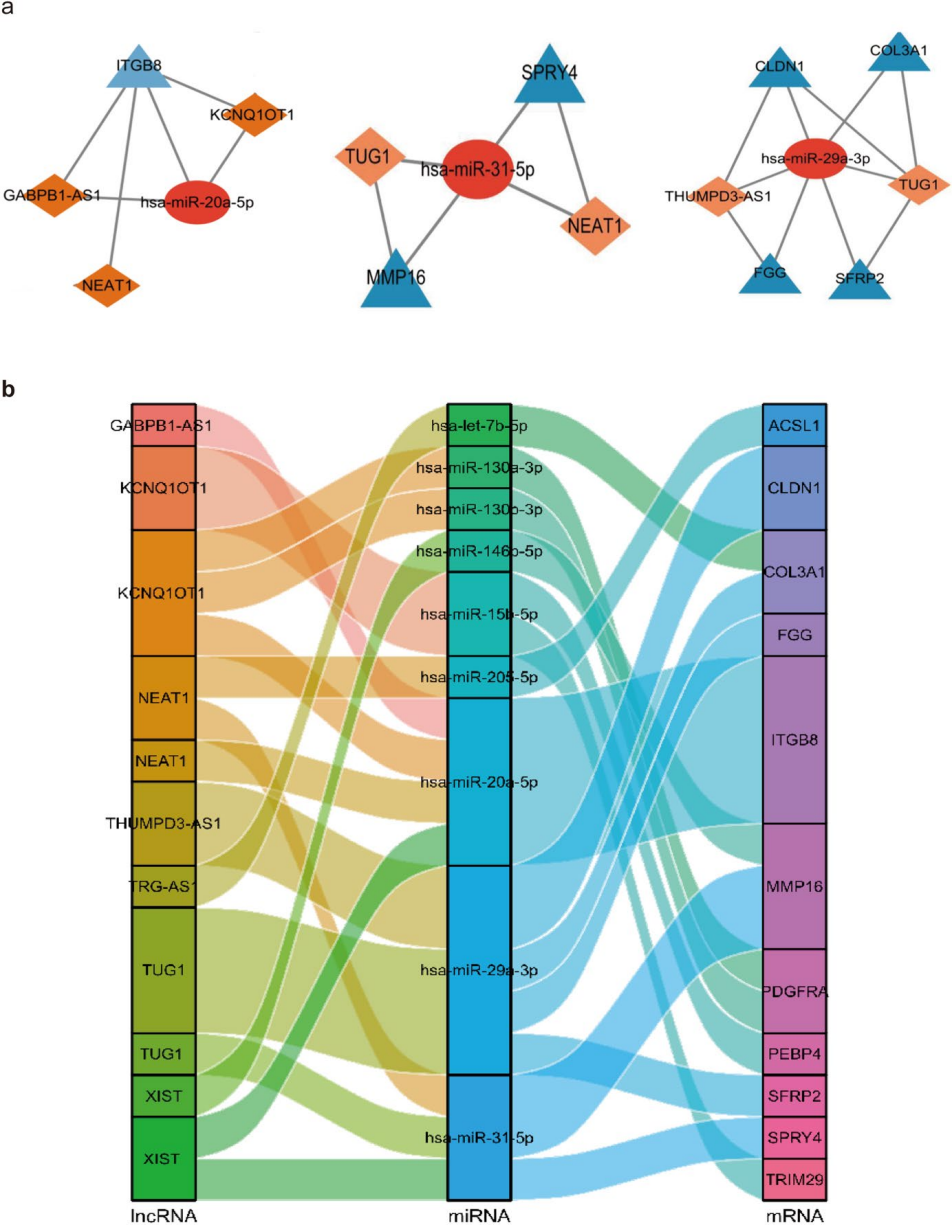
**a** Ellipses represent miRNAs, triangles represent mRNAs, and rhomboids represent lncRNAs. **b** The Sankey plots of the ceRNA networks. Results of enrichment analysis of candidate miRNAs. (*ceRNA* competing endogenous RNA)

In this study, DEmiRNAs were used to screen miRNA-targeted mRNAs in the miRTarBase database. The identified miRNA-targeted mRNAs were compared with DEmRNAs previously identified in the GSE32537 and GSE47460 datasets. This comparison led to the identification of 14 candidate mRNAs (Fig. 5a). GO and KEGG pathway enrichment analyses indicated that the candidate mRNAs were significantly associated with several BP, such as extracellular structure organization and cell–matrix adhesion. Enrichment of MFs was observed in various binding activities, such as binding with cell adhesion molecules. Furthermore, the analysis revealed enriched CC in the collagen-containing extracellular matrix, endoplasmic reticulum lumen, blood microparticles, platelet alpha granules, and platelet alpha granule lumens (Fig. 5b). Additionally, KEGG pathway analysis demonstrated significant enrichment of DEmRNAs in four pathways: platelet activation, Wnt signaling, cell adhesion molecules, and complement and coagulation cascades (Fig. 5c). Furthermore, the interaction between DEmiRNAs and lncRNAs was predicted using StarBase and lncBase v3.0 databases. The results of this prediction were then cross-referenced with the DElncRNAs to obtain a list of candidate lncRNAs (Fig. 5d, e). Candidate miRNA-mRNA and miRNA-lncRNA networks were screened for ceRNA networks using the lncACTdb database. The DEmiRNAs, mRNAs, and predicted lncRNAs involved in the ceRNA network are presented in Table 5. For instance, lncRNAs such as KCNQ1OT1, XIST, and NEAT1 are involved in different ceRNA regulatory axes, and the same mRNA is regulated by multiple lncRNAs, including ITGB8, MMP16, and COL3A1. Specific ncRNAs were associated with the following ceRNA regulatory axes: KCNQ1OT1-miR-130a-3p/miR-130b-3p-PDGFRA, KCNQ1OT1-miR-15b-5p-PEBP4/TRIM29, KCNQ-1OT1/XIST/NEAT1/GABPB1-AS1-miR-20a-5p-ITGB8, XIST-miR-146b-5p/miR-31-5p-MMP16, NEAT1-miR-205-5p-ACSL1, THUMPD3-AS1/TUG1-miR-29a-3p-COL3A1, TRG-AS1-let-7b-59-COL3A1, and NEAT1/XIST-miR-31-5p-MMP16/SPRY4.To visually represent the results, a Cytoscape network and Sankey plots were constructed (Fig. 6a-b).

**Table 5:** The DEmiRNAs, DEmRNAs, and predicted lncRNAs in the ceRNA network

miRNA	mRNA	lncRNA
hsa-let-7b-5p	COL3A1	TRG-AS1
hsa-miR-130a-3p	PDGFRA	KCNQ1OT1
hsa-miR-130b-3p	PDGFRA	KCNQ1OT1
hsa-miR-146b-5p	MMP16	XIST
hsa-miR-15b-5p	PEBP4, TRIM29	KCNQ1OT1
hsa-miR-205-5p	ACSL1	NEAT1
hsa-miR-20a-5p	ITGB8	GABPB1-AS1, KCNQ1OT1, NEAT1, XIST
hsa-miR-29a-3p	CLDN1, FGG, SFRP2, COL3A1	THUMPD3-AS1, TUG1
hsa-miR-31-5p	MMP16, SPRY4	NEAT1, TUG1, XIST

*miRNA* microRNA, *DEmiRNA* differentially expressed miRNA, *mRNA* messenger RNA, *DEmiRNA* differentially expressed mRNA, *lncRNA* long non-coding RNA, *DELncRNA* differentially expressed lncRNA, *ceRNA* competitive endogenous RNA

### Enrichment Analysis of Candidate DEmiRNAs

**Fig. 7:**
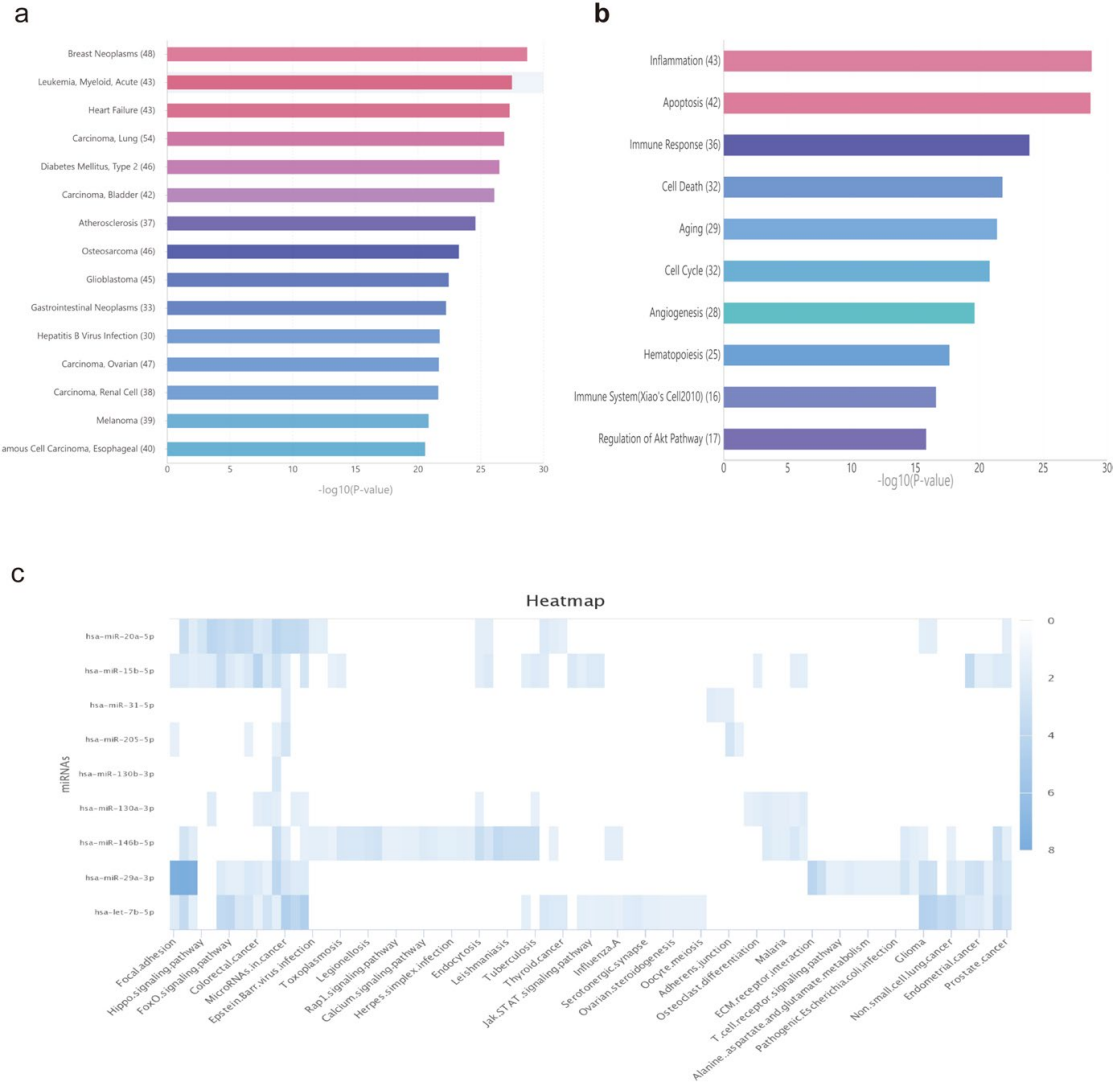
**a** and **b** Top significantly enriched GO terms of candidate DEmiRNAs, including diseases and MFs. **c** Heatmap of the results of KEGG pathway analysis of candidate DEmiRNAs. (*GO* Gene Ontology, *MF* molecular functions, *KEGG* Kyoto Encyclopedia of Genes and Genomes)

The results of GO and KEGG pathway enrichment analyses demonstrated a significant association between the candidate DEmiRNAs involved in the ceRNA network and 15 diseases, including breast neoplasms, acute myeloid leukemia, heart failure, and lung carcinoma (Fig. 7a). Additionally, the candidate DEmiRNAs were significantly enriched in 10 MF, including inflammation and apoptosis (Fig. 7b). KEGG pathway analysis further revealed that the candidate DEmiRNAs were primarily enriched in the focal adhesion, Hippo signaling, and FoxO signaling pathways (Fig. 7c).

### Correlation Analysis Between the Expression of Candidate DEmRNAs and %Predicted FVC (pre-bd)

**Fig. 8:**
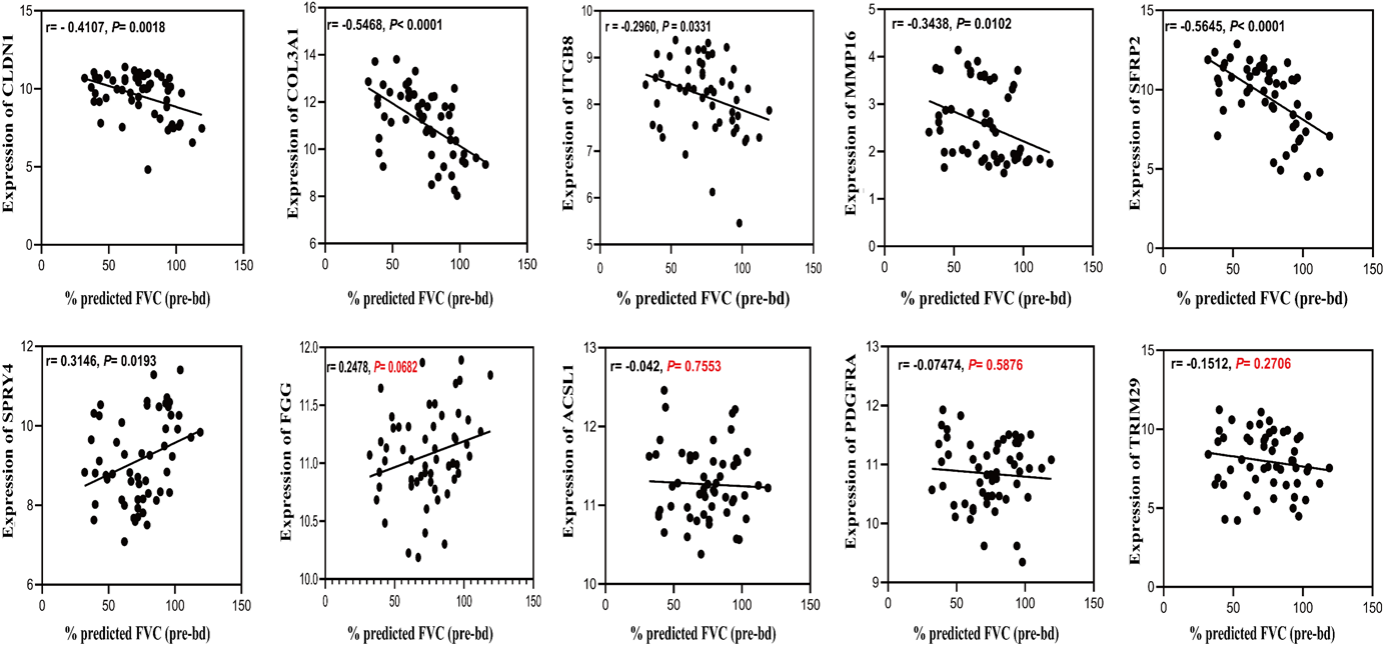
Correlation between the expression of candidate mRNAs and %predicted FVC (pre-bd). (%predicted FVC (pre-bd): % predicted pre-bronchodilator forced vital capacity)

To evaluate the clinical significance of the DEmRNAs, we analyzed to determine whether there was a relation between the expression levels of these DEmRNAs and the %predicted FVC (pre-bd). This analysis used the clinical information available in the GSE47460 database and the gene expression data of candidate DEmRNAs. The findings revealed that the expression of CLDN1, COL3A1, MMP16, SFRP2, SPRY4, and ITGB8 correlated with the %predicted FVC (pre-bd). Specifically, SPRY4 expression was positively correlated with %predicted FVC (pre-bd), whereas the remaining genes were negatively correlated with %predicted FVC (pre-bd). No significant correlation was observed between the expression of ACSL1, FGG, PDGFRA, and TRIM29 and %predicted FVC (pre-bd) (Fig. 8).

### Validation of Candidate DEmRNAs Using qRT-PCR

**Fig. 9:**
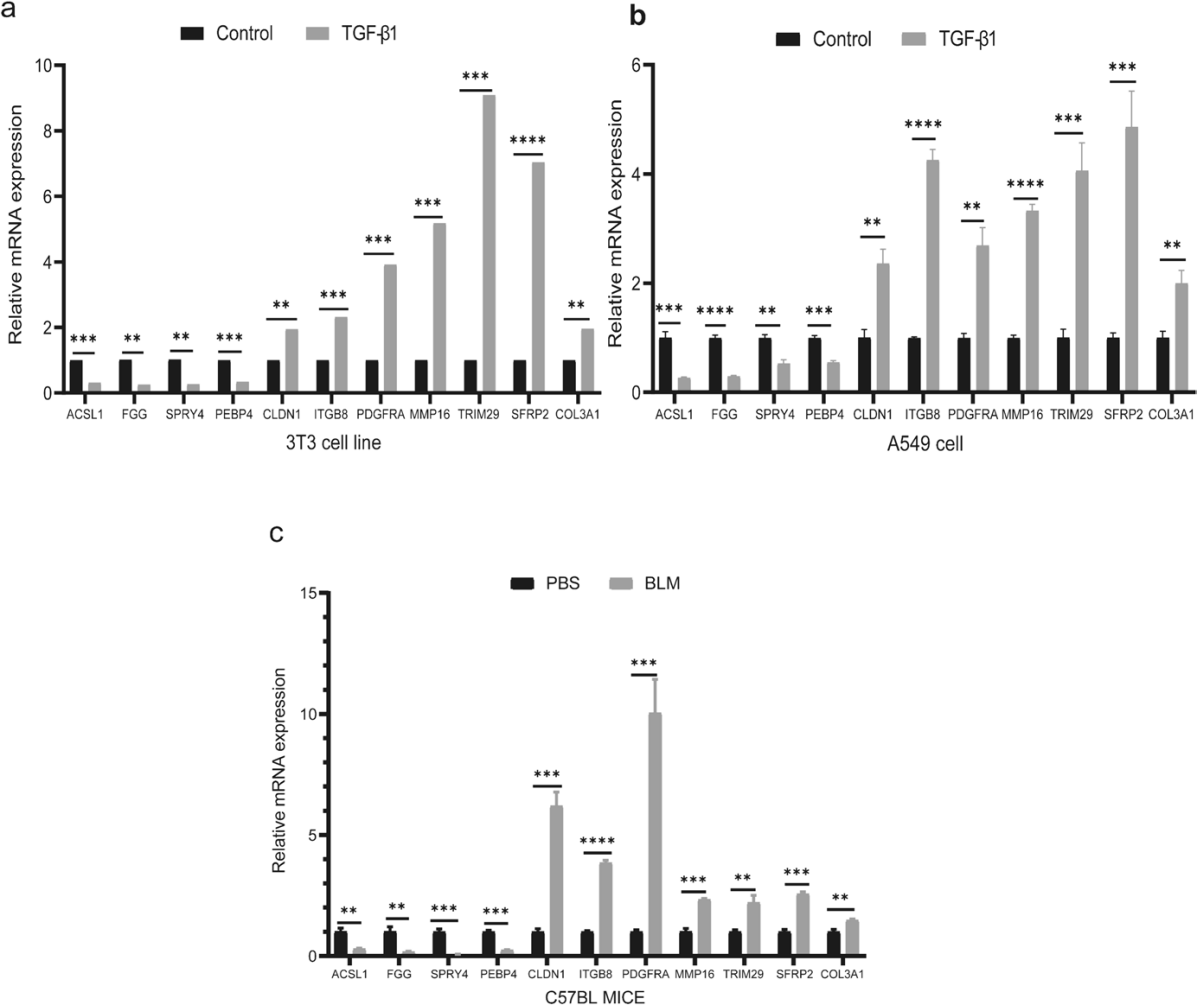
Expression levels of candidate DEmRNAs were assessed using qRT-PCR. (a and b): In 3T3 and A549 cells, the expression of ACSL1, FGG, SPRY4, and PEBP was downregulated in the TGF-β1 group compared with the Control group, while the expression of CLDN1, ITGB8, PDGFRA, MMP16, TRIM29, SFRP2, and COL3A1 was upregulated. (c): In C57BL mice, the expression of ACSL1, FGG, SPRY4, and PEBP was downregulated in the BLM group compared with the PBS group, while the expression of CLDN1, ITGB8, PDGFRA, MMP16, TRIM29, SFRP2, and COL3A1 was upregulated. (**P < 0.01, *** P < 0.001, **** P < 0.001). (*qRT-PCR* quantitative real-time reverse transcriptase polymerase chain reaction, *PBS* phosphate-buffered saline, *BLM* bleomycin)

In this study, we used qRT-PCR to evaluate the expression of candidate DEmRNAs in 3T3 and A549 cells following activation induced by TGF-β1. Our findings revealed that the expression levels of ACSL1, FGG, SPRY4, and PEBP were significantly decreased in the TGF-β1 group compared with the control group. Conversely, the expression levels of CLDN1, ITGB8, PDGFRA, MMP16, TRIM29, SFRP2, and COL3A1 were significantly increased (Fig. 9a-b). Similar trends were observed in the BLM-treated mice (Fig. 9c).

## Discussion

IPF is a highly lethal and rapidly progressive disease with increasing incidence worldwide. Its occurrence is now comparable to that of cancers, such as gastric, liver, testicular, and cervical cancers (Hutchinson et al. 2015). Early diagnosis and treatment options for IPF are limited, and there is currently no known cure. Therefore, it is crucial to identify specific diagnostic markers and therapeutic targets in patients with IPF. In recent years, there has been a growing recognition of the role of ncRNAs in the development of various diseases, including IPF. It has been discovered that lncRNAs can act as ceRNAs in the pathogenesis of IPF. The ceRNA mechanism establishes a connection between mRNAs and ncRNAs that encode proteins, thereby influencing the occurrence and progression of IPF. However, the role of lncRNA-mediated ceRNA networks in the pathogenesis of IPF has not been well studied, and the detailed mechanism requires further exploration. Therefore, to elucidate the potential pathogenesis of IPF, we constructed a ceRNA regulatory network based on four microarray datasets.

This study identified seven candidate DElncRNAs. Among them, TRG-AS1, THUMPD3-AS1, and GABPB1-AS1 have not been studied in fibrotic diseases but have been documented in other conditions such as lung cancer, gastric cancer, and osteosarcoma. These lncRNAs have been shown to regulate cell proliferation, invasion, epithelial-mesenchymal transition, and inflammatory response (Chen et al. 2022; Zhang et al. 2021b, 2023). Additionally, KCNQ1OT1, XIST, NEAT1, and TUG1 were found to be associated with multiple ceRNA regulatory networks, potentially exerting crucial roles in the pathophysiology of idiopathic pulmonary fibrosis (IPF) by affecting numerous target genes and participating in different biological processes or signaling pathways. While there are reports of these DElncRNAs in fibrotic diseases, for instance, Yang et al. demonstrated that KCNQ1OT1 regulates lung fibrosis by modulating Rtn3 expression in a lipopolysaccharide-induced acute lung injury mouse model, the specific pathways and cellular functions involved in this process require further investigation (Yang et al. 2022).Similarly, Wang et al. reported that XIST influences lung fibrosis development by modulating β-catenin expression through miR-139(Wang et al. 2017b). However, validation of these gene regulatory molecules in IPF patient lung tissues and their correlation with clinical features remain to be explored comprehensively. Regarding NEAT1, Zhang et al. reported that NEAT1 positively regulates NFATc3 expression by directly targeting miR-29a (Zhang et al. 2022), but this finding was only validated in cellular models and requires in vivo confirmation. These findings underscore the potential indispensable role of these DElncRNAs in the overall regulatory network of IPF. Given the limited research on related regulatory networks, exploring the regulatory mechanisms of these three candidate DElncRNAs is particularly important. Screening of ceRNA networks can provide clues to their regulatory networks.

For the candidate DEmRNA molecules in the ceRNA network, we validated their expressions and analyzed their correlation with the predicted FVC (pre-bd) % to determine whether these candidate DEmRNAs have clinical significance. The %predicted FVC (pre-bd) is a crucial indicator for assessing lung function and disease progression in IPF patients. Our results indicate differential expression of these DEmRNA molecules, among which the expressions of CLDN1, COL3A1, MMP16, SFRP2, SPRY4, and ITGB8 correlate with % predicted FVC (pre-bd). Interestingly, we found that MMP16 and ITGB8 are also involved in multiple ceRNA regulatory networks. Does this imply that these two mRNAs may be key factors in IPF regulation? ITGB8, a transmembrane heterodimer, serves as the primary receptor for extracellular matrix (ECM) adhesion and recognition (Takada et al. 2007). Integrin exhibits sustained activation in abnormally activated lung fibroblasts, leading to fibrosis progression. Downregulation of ITGB8 expression significantly alleviates fibrosis in the liver, lungs, and kidneys (Bagnato et al. 2018; McCarty 2020). For instance, Minagawa et al. confirmed that inhibiting TGF-β activation using avβ8 antibodies effectively prevents fibroblast activation in the airways of patients with chronic obstructive pulmonary disease (Minagawa et al. 2014). MMP16 belongs to the calcium-zinc-dependent metalloproteinase family, responsible for ECM maintenance. It plays a crucial role in various pathological processes such as atherosclerosis, tumor invasion, and fibrosis (Pittayapruek et al. 2016). Studies have shown higher expression of MMP16 in allergic asthma mice compared to normal mice, and regulating MMP16 expression can reduce fibrosis (Zhong et al. 2022). IPF is characterized by excessive extracellular matrix deposition, highlighting the significance of in-depth research on ITGB8 and MMP16 in ECM regulation. However, current research on this topic is limited, and the specific regulatory pathways and involved biological processes remain unknown. With the construction of ceRNA networks, new clues can be provided for their upstream regulatory relationships, and further investigation into regulatory mechanisms can offer new insights for targeted IPF therapy.

Based on the aforementioned data, it can be inferred that the ceRNA network containing lncRNA KCNQ1OT1, XIST, and NEAT1, as well as mRNA ITGB and MMP16, may play a crucial regulatory role in the development of IPF. These networks include hsa-miR-146b-5p, hsa-miR-20a-5p, and hsa-miR-31-5p. Hsa-miR-20a-5p is a member of the has-mir-17 cluster. Studies have indicated a reduction in the expression of miR-20a in IPF patients, while the miR-17 ~ 92 cluster is associated with lung fibrosis development (Dakhlallah et al. 2013). However, the specific regulatory mechanisms remain unclear. Another miRNA, hsa-miR-146b-5p, is downregulated in lung tissue samples from IPF patients. Subsequent studies by Shuai et al. found that miR-146b-5p can inhibit lung fibrosis through the Notch1/PDGFRβ/ROCK1 pathway (Mullenbrock et al. 2018). However, the reliability and reproducibility of this mechanism are compromised due to imperfect experimental design. Although hsa-miR-31-5p has not been specifically studied in the context of lung fibrosis, it is highly expressed in hypertrophic scar fibroblasts. Knocking out miR-31-5p significantly inhibits fibroblast proliferation under hypoxic conditions, promotes cell invasion, and suppresses the expression of collagen I, collagen III, and fibronectin (Wang et al. 2017a). Therefore, investigating the role of these miRNAs in fibrotic diseases is worthwhile.

However, this study has several limitations. First, it relies solely on data from the GEO database. Although four different IPF microarray datasets were integrated, the sample size remained limited, which may have affected the statistical significance and generalizability of the findings. Second, there was a lack of validation of the expression levels of the identified candidate DEmiRNAs and DElncRNAs and a lack of validation of the expression levels of candidate DEmRNAs in human lung tissues. Additionally, the assessment of the clinical value of the candidate DEmRNAs was based solely on correlation analysis without any validation. Overall, this study is still in the exploratory stage, and the underlying regulatory mechanisms of the ceRNA network have not been fully elucidated through the observed level changes and bioinformatics analyses. Further functional biology experiments with larger sample sizes are required to validate these mechanisms.

## Conclusion

This study has constructed three ceRNA regulatory networks (KCNQ1OT1/XIST/NEAT1-miR-20a-5p-ITGB8, XIST-miR-146b-5p/miR-31-5p-MMP16, and NEAT1-miR-31-5p-MMP16). These findings contribute to a deeper understanding of the pathogenesis of IPF and provide clues for identifying potential therapeutic targets and further research directions.

## Data Availability

The row data included in this study are available in GEO [https://www.ncbi.nlm.nih.gov/geo/, (accessed on 25 May 2022)].
